# Panning for Long Noncoding RNAs

**DOI:** 10.3390/biom3010226

**Published:** 2013-02-28

**Authors:** Shanshan Zhu, Xiao-Ou Zhang, Li Yang

**Affiliations:** Key Laboratory of Computational Biology, CAS-MPG Partner Institute for Computational Biology, Chinese Academy of Sciences, Shanghai 200031, China; E-Mails: zhushanshan@picb.ac.cn (S.Z.); zhangxiaoou@picb.ac.cn (X.-O.Z.)

**Keywords:** long noncoding RNA (lncRNA), computational analysis, deep sequencing, transcriptome

## Abstract

The recent advent of high-throughput approaches has revealed widespread transcription of the human genome, leading to a new appreciation of transcription regulation, especially from noncoding regions. Distinct from most coding and small noncoding RNAs, long noncoding RNAs (lncRNAs) are generally expressed at low levels, are less conserved and lack protein-coding capacity. These intrinsic features of lncRNAs have not only hampered their full annotation in the past several years, but have also generated controversy concerning whether many or most of these lncRNAs are simply the result of transcriptional noise. Here, we assess these intrinsic features that have challenged lncRNA discovery and further summarize recent progress in lncRNA discovery with integrated methodologies, from which new lessons and insights can be derived to achieve better characterization of lncRNA expression regulation. Full annotation of lncRNA repertoires and the implications of such annotation will provide a fundamental basis for comprehensive understanding of pervasive functions of lncRNAs in biological regulation.

## 1. Introduction

It is well known that DNA is transcribed into messenger RNA (mRNA), which is then translated to protein(s) with the help of housekeeping noncoding RNAs (ncRNAs) such as transfer RNAs (tRNAs) and ribosomal RNAs (rRNAs). Messenger RNAs serve as intermediate carriers, forwarding genetic information (as coding genes) from DNA to protein. Characterization of coding genes and their protein products has been of great importance in our goal to understand gene expression regulation. While early expectations were to find about 100,000 genes in the human genome, the current estimate stands at 20,000-25,000 [1] genes after the first draft of the human genome was released in 2001 [2]. We have now learned that only about 2% of the human genome encodes protein sequences [1], much of the rest of the noncoding segments used to be considered as “junk” or “dark matter” [3,4], despite evidence of their participation in gene expression regulation at multiple levels. Housekeeping ncRNAs with known functions have been studied for many decades. For example, they play key roles in translation (tRNA and rRNA), splicing (snRNA), and RNA modification (snoRNA). The advent of state-of-the-art deep sequencing technology has revealed that most of the human genome is pervasively transcribed [5,6], indicating a rich pool for ncRNAs besides the aforementioned well characterized molecules. 

New small regulatory ncRNAs were first identified by exogenous RNA interference in plants and nematodes, and later found to exist endogenously. These small ncRNAs, including but not limited to microRNAs (about 22 nt long), function as posttranscriptional repressors [7]. Through a combination of size selected high-throughput sequencing and computational approaches, a very large number of small ncRNAs have now been identified and predicted in genomes, and their evolutionary conservation and structural stability have been extensively analyzed [8]. Generally speaking, the computational pipeline for small ncRNA prediction with high-throughput experiments is now relatively mature [9,10], and over 1600 precursors and 2042 mature miRNAs have been reported in the human genome (miRBase 19, released date August 2012).

Beyond the small regulatory ncRNAs, the multifaceted transcriptome has become even more complex with the discovery of the pervasive transcription of long noncoding RNAs (lncRNAs, at least 200 nt long). LncRNAs are known to play important roles in both biological and pathological events [11,12,13,14], including X-chromosome inactivation (*Xist*) [15], genomic imprinting (*Air*, *Kcnq1ot1*) [16,17] and nuclear trafficking (*NORN*) [18]. The application of tiling arrays allowed the discovery of additional lncRNAs, including the well-characterized *HOTAIR* [19], *NEAT1* and *MALAT1* [20]. These lncRNAs are involved in *trans*-acting gene regulation (*HOTAIR*) [19], providing a structural scaffold in nuclear architectures (*NEAT1*) [21,22,23,24] and alternative splicing regulation (*MALAT1*) [25], although the effects might be very subtle as indicated by discrepancies in cell cultures [25] and mouse models [26]. Detailed studies of these abundant lncRNAs have served as road maps for the functional characterization of other lncRNAs. Very recently, the new finding and understanding of pervasive transcription from the “dark matter” attracted our attention to an integrated annotation of lncRNAs from transcriptomes. The existence of thousands of lncRNAs from intergenic regions (large intergenic noncoding RNA, lincRNA) has been inferred from massive high-throughput sequencing data including histone modification landscapes (chromatin signatures) in both mouse [27] and human [28]. In addition, functional investigations of certain lncRNAs further revealed additional roles of these molecules in gene expression regulation, from controlling chromatin complexity [29], to acting as competing endogenous RNAs [30], to performing enhancer-like functions [31], and to maintaining pluripotency [32] and embryogenesis [33]. In addition, non-polyadenylated RNA enrichment from human transcriptomes, followed by computational analysis, revealed that some excised introns can stably accumulate as lncRNAs [34]. In some cases, intron-derived lncRNAs are capped by snoRNAs at both ends to protect intronic sequences from degradation after splicing, leading to the formation of a new class of lncRNAs (*sno-lncRNA*s) [35]. 

As up to 70% of the human genome can be transcribed [36] and only about 2% of the human genome encodes protein coding genes including UTRs [1], it is not surprising that the majority of lncRNAs were previously classified as “junk sequences” or “dark matter”. Some of the best-characterized lncRNAs are generally highly expressed and conserved across species, but these features are more the exception than the rule and cannot be generalized to thousands of other lncRNAs identified by large-scale screening. The latter are generally expressed at a low level [37] and are less conserved [38], which have impeded their discovery and functional studies. In this review, we assess issues that have challenged lncRNA discovery in the past, and also highlight recent experimental and computational designs that have facilitated lncRNA identification and characterization. These advances not only shed light on lncRNA characterization but also reveal the complex mechanisms they use to regulate other molecules. 

## 2. Challenges for LncRNA Discovery

**Figure 1:**
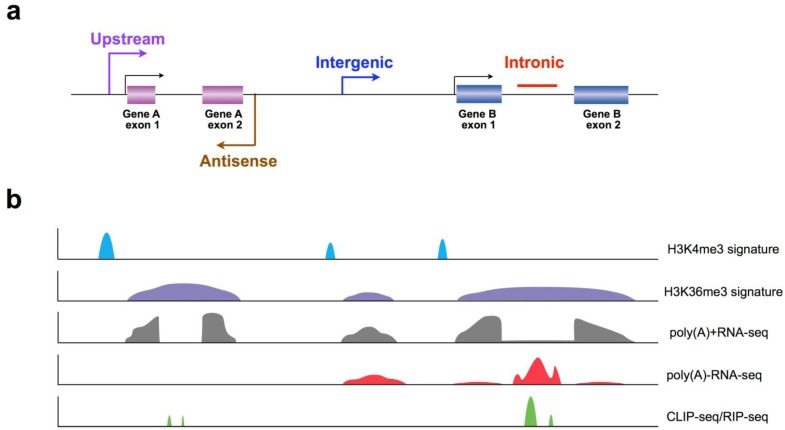
Schematic diagram of long noncoding RNA discovery and function analysis using genome-wide methods. (*a*) Genomic locations for long noncoding RNA (lncRNA) transcription. Boxes shown as annotated genes and exons. Arrows label the direction of transcription. (*b*) Methodology for lncRNA discovery and functional association with proteins. H3K4me3 signature defines transcription initiation. H3K36me3 signature defines transcription elongation. Signals of poly(A)+RNA-seq indicate polyadenylated RNAs (including most annotated mRNAs and lncRNAs). Signals of poly(A)-RNA-seq indicate non-polyadenylated RNAs, including recently identified intronic transcripts. Signals of CLIP-seq/RIP-seq reveal the association of RNA transcripts with RNA binding proteins.

In the first decade of this century, whole genome sequencing revealed approximately 20,000 protein coding genes in humans, which is comparable to estimates in the fly and worm, although humans exhibit much more complexity through alternative splicing [1,2]. With the rapid development of high-throughput technologies, growing lines of evidence have indicated that genomes are pervasively transcribed, with many previously ignored portions of the genome transcribed as lncRNAs [6,36,38] (Figure 1*a*). However, several intrinsic features of lncRNAs have posed challenges for their discovery as well as their functional study, as discussed below.

### 2.1. LncRNAs in General Are Expressed at Low Levels *in vivo*, but with High Tissue-Specificity

RNA-seq (deep sequencing from reverse-transcribed RNAs) datasets revealed that the human genome is pervasively transcribed [5]. However, the extent of this pervasive transcription has been disputed [39,40]. The controversy has been partially due to different datasets and computational approaches [6] that were applied to individual analyses, but also to the nature of the low expression in most noncoding regions in genomes. For example, many such transcripts from intergenic or intronic regions were detected at very low levels by various technologies [41]. In addition, the median expression level of lincRNAs was approximately one-third of that of the coding ones in the mouse [42] and about 10-fold lower than of coding genes in humans [28]. Moreover, the recent Encyclopedia of DNA Element (ENCODE) project released a variety of transcriptomes of RNA repertoires from 15 human cell lines. The complete annotation of these transcriptomes suggested that lncRNAs have lower expression levels than coding RNAs [36]. In particular, 80% of detected lncRNAs exist in ≤1 copy per cell, compared with only 25% of coding RNAs in examined cell lines [36]. Taken together, the nature of low expression of lncRNAs makes it difficult for their discovery, precise annotation, and subsequent functional studies. Nonetheless, the expression of a few lncRNAs is comparable or even higher than coding ones in certain cell lines (e.g., *H19* in NHEK cells [36] and *sno-lncRNA*s in hES cells [35]). 

Accumulated results suggested that most lncRNAs exhibit a low level of expression but high tissue-/cell-specific patterns [37,38,43]. About 78% of human lincRNAs are tissue-specific, compared with about 19% for protein coding genes [28]. Moreover, the complete transcriptome analyses from 15 human cell lines in the ENCODE project showed that 29% of all detected lncRNAs are only from one cell line and only 10% are expressed in all cell lines. In contrast, 7% of expressed coding RNAs were only detected from one cell line, but 53% of them were expressed in all cell lines [36]. These observations indicate that their tissue-specific expression patterns make the identification and characterization of these lncRNAs quite challenging if only a small portfolio of tissues/cell lines are chosen for analyses. 

### 2.2. Evolutionary Conservation of LncRNAs on Average Is Relatively Lower than That of Coding RNAs

Homologous sequence comparison is an efficient method for identifying genes that exhibit similar functions between species and for discovering novel coding regions [44], however, it is not an effective way for non-protein coding sequences, because they are less conserved. For example, only a small portion (<5%) of noncoding sequences are conserved between human and mouse [5,45]. Recent transcriptome analyses by a variety of RNA-seq experiments indicated the existence of thousands of lowly conserved lncRNAs from zebrafish [46] genome to mouse [27] and human [28] genomes. Only 29 out of 550 lincNRAs in zebrafish have detectable sequence similarity with putative mammalian orthologs, and similar sequences are typically restricted to a single short region of high conservation [46]. Thus, although lncRNAs are less conserved across species than protein coding genes, they still on average represent somewhat higher levels of conservation than random regions or introns [42].

Usually, evolutionary constraint can be estimated from the nucleotide substitution rate in functional sequences [47]. Nucleotide substitutions in ncRNAs are on average about 90-95%, compared with about 10% in coding genes. This is reasonable, as nucleotide substitutions tend to be less deleterious in noncoding sequences than in coding ones [47]. A limited phylogenetic range of ncRNAs can be explained as emerging or declining rapidly within particular lineages [48]. For instance, it has been suggested that about one third of lncRNAs have arisen within the primate lineage only [38]. 

The aforementioned studies suggested that low evolutionary conservation might be a natural feature of noncoding transcripts, which is consistent with their rather poor genome-wide annotations in early studies [1,2,4]. However, considering the relatively higher species divergence, it is possible to identify more novel lncRNAs from different species/evolutionary lineages. Their generally low expression level together with poor conservation initially led researchers to conclude that transcripts from noncoding segments may represent transcriptional noise [49]. However, lack of conservation does not mean lack of function [50]. For example, human *NEAT1* RNA and its mouse homolog *Men* *ε/β* have low sequence similarity [20] but are functionally conserved [21,22,23,24]. Interestingly, some mouse pseudogenes, whose ancestors have lost their protein-coding capabilities during rodent evolution, have retained their expression and act as competitive noncoding RNAs and function as miRNA-decoys [51]. In fact, an increasing number of intensive functional studies have shown that lncRNAs are not just ancient relics with little function, but have a variety of roles from epigenetic regulation to pluripotency maintenance, and are also highly correlated with some human diseases [52,53].

### 2.3. Controversial Coding Capacity of LncRNAs

Exclusion of protein-encoding capacity is a fundamental requirement for lncRNA definition. In the post-genomic era, this capacity can be predicted genome wide using computational approaches, mainly based on the length and conservation of ORFs [54]. Cutoffs for minimal ORF length, if applied for 300 nt (100 amino acids) [55] or even 60 nt (20 amino acids) [56], can still cause controversy. For example, some well-characterized lncRNAs, such as *Xist* [57], have remnants featuring longer-than-100-amino acid ORFs. With widespread transcription from a given genome, one can imagine that many transcripts identified as lncRNAs may contain ORF remnants, while some coding RNAs may contain only small ORFs for short polypeptides. In this case, computational algorithms with multiple features incorporated are needed to distinguish truly noncoding RNAs from coding ones. For instance, CPC [58] contains six features to not only evaluate the extent and quality of ORFs, but also parse the ORF conservation of sequences using BLASTX [59]. Although low conservation of ORFs reflected the gene evolution in specific lineages or gene loss in other lineages, studies suggested that most putative human ORFs with no cross-species counterparts are likely to be random occurrences [60] and this is indeed the case for *Xist* [57]. A phylogenetic model of codon substitution frequency (phyloCSF) metric by orthologous transcript comparison was chosen to distinguish noncoding transcripts from coding ones [61], and successfully applied for lincRNA predictions in both mouse [42] and humans [28]. 

Besides computational judgments based on critical features of putative ORFs, several other crucial criteria, such as the subcellular localization and the accessibility to the translation machinery, could also be used to evaluate whether a given transcript is a true lncRNA or not. RNA transcripts localized in the nucleus principally suggest functions that are primarily non-coding. This can be estimated experimentally by RNA fractionation from nuclear homogenates [38], as exemplified by *NEAT1* [21] and *DEB-T* [62], despite the risk of possible nuclear/cytoplasmic leakage during RNA isolation. RNA fluorescence *in situ* hybridization (FISH) is an alternative way to examine the subcellular localization. A growing list of well-characterized lncRNAs do localize in the nucleus and within specific subnuclear structures as illuminated by RNA FISH and are associated with nuclear proteins as revealed by RNA-protein double FISH [63]. Furthermore, ribosome profiling coupled with RNA-seq can provide extra insights for the accessibility of a given transcript to the translational machinery [64]. Moreover, proteome datasets with a spectrum of all protein products can also be applied to mine the existence/non-existence of coding products from tested transcripts. These datasets offer the most direct evidence to determine coding capacity of any transcript, although with low resolution and low availability. Finally, it cannot be ruled out that some transcripts have a dual nature, acting both as ncRNA and producing protein products [65,66].

The best way to distinguish between coding and non-coding sequences is to integrate computational and experimental approaches that enhance understanding of lncRNA expression regulation and biological function *in vivo*.

## 3. Recent Progress in LncRNA Discovery Using New Strategies

With technological improvements and the application of integrated methodologies, significant progress has been achieved in uncovering new lncRNA molecules. Some of these practical strategies can be further applied to achieve new insights into lncRNA functions. 

### 3.1. Application of Chromatin Signatures to Determine LncRNAs from Intergenic Regions

Several individual studies have applied a systematic and integrative strategy with multiple biological features to identify lncRNAs, mainly in intergenic regions (lincRNAs), first in mouse [27] and then in zebrafish [46] and human [28] genomes. Distinguished from other previous trials, a brand new feature of “H3K4me3-H3K36me3” chromatin signatures has been utilized in all three species to confirm lncRNA promoters using the histone 3 Lys 4 trimethylation (H3K4me3) signature followed by identification of actively transcribed lncRNA regions using the histone 3 Lys 36 trimethylation (H3K36me3) signature. By differentiating the “H3K4me3-H3K36me3” chromatin signatures of lncRNAs from those of known coding genes/microRNAs/endogenous siRNAs, these analyses reliably identified lncRNA-expressed genomic sequences, largely in intergenic regions (Figure 1*b*). In addition, other stringent criteria have also been taken into account for lncRNA characterization, including the identification of poly(A) sites, transcription initiation signals, expression patterns among tissues and potential coding capacity. Loss-of-function and gain-of-function of certain conserved lncRNAs demonstrated crucial biological roles of lncRNAs in zebrafish [46], indicating functional conservation despite limited sequence conservation. More importantly, 7some lincRNAs have been shown to play important roles in multiple layers of biological processing, including epigenetic regulation and pluripotency maintenance (reviewed by Guttman [14], Rinn [13] and their colleagues).

### 3.2. Development of a Non-Polyadenylated RNA Enrichment Strategy to Uncover LncRNAs from Introns

Most RNA polymerase II transcripts, including mRNAs and lncRNAs, are polyadenylated (poly(A)+) at their 3’ ends. The application of transcriptome analysis of poly(A)+ RNA by high-throughput deep sequencing (mRNA-seq) has revealed a digital map of poly(A)+ transcripts from both known and previously un-annotated genes [67]. However, the transcribed portion of the genome is more than poly(A)+ transcripts, and there are a large number of non-polyadenylated transcripts (poly(A)− transcripts), including ribosomal RNAs (rRNAs) generated by RNA polymerases I and III, other small RNAs generated by RNA polymerase III, replication-dependent histone mRNAs [68] and some lncRNAs [24,69] transcribed by RNA polymerase II. Depletion of ribosomal RNAs (RiboMinus) from total RNA results in both poly(A)+ and poly(A)− transcripts available for deep sequencing analysis. This has led to the discovery of many new poly(A)− transcripts when compared with poly(A)+ RNA deep sequencing [70,71]. However, rRNA-depletion methods cannot physically separate poly(A)− transcripts from poly(A)+ RNAs, thus it is difficult to directly annotate poly(A)− transcripts using only the rRNA-depletion method. Recently, a combination of both rRNA and poly(A)+ RNA removal was applied to obtain a largely pure population of poly(A)- RNAs for high-throughput deep sequencing [34]. This type of poly(A)− RNA-seq of the human cell transcriptomes surprisingly revealed many previously un-annotated RNA transcripts, including a new family of lncRNAs from introns in humans [35] (Figure 1*b*). In addition, with the same separation strategy for poly(A)− transcripts followed by deep sequencing analyses, additional poly(A)− lncRNAs from intronic regions were also found in various human cell lines [38]. Interestingly, RNA fractionation from nuclear homogenates also indicated the presence of stable intronic sequence RNAs in *X. tropicali*s [72]. As most lncRNAs are tissue/cell-specific and species-specific, further application of poly(A)− RNA-seq for different tissues and species may result in the identification of additional intron-derived lncRNAs.

What mechanism(s) can generate RNA transcripts without canonical poly(A) tails at their 3' ends? For most of the replication-dependent histone pre-mRNAs, evolutionarily conserved stem-loop structures in their 3’ UTRs direct U7 snRNA-mediated 3’ end formation to stabilize mature mRNAs and confer cell cycle dependent regulation of their accumulation [67]. For *MALAT1* and *Menε/β* lncRNAs, their 3' end maturation depends on RNase P cleavage [24,69], stabilized by highly conserved A- and U-rich motifs that form a triple-helical structure [73,74]. For telomerase RNA in *S. pombe*, incomplete splicing, but not the complete splicing, generates a functional *TER1* transcript [75]. However, it appears that none of the above mechanisms are applicable to explain the biogenesis of lncRNAs from introns, as introns are generally rapidly degraded after splicing. Yin *et al* recently demonstrated that intron-derived *sno-lncRNA*s depend on the snoRNA machinery at both ends for their processing and on snoRNP complexes at both ends to protect intronic sequences from exonucleotic trimming [35]. Genome-wide analysis of poly(A)− RNAs from introns has revealed a large number of lncRNAs from intron regions [34,38]; however, only some are capped with snoRNAs. The biogenesis of others needs to be further addressed. Finally, in addition to poly(A)− RNA-seq, the development of more specific experimental and computational approaches will help to understand other poly(A)− lncRNAs matured by RNase P cleavage or incomplete splicing.

### 3.3. Determination of Co-Factors to Study LncRNA Biogenesis and Function

It’s now clear that lncRNAs play important roles in a variety of biological processes [13,14,63]. So far, only a handful of mechanisms have been identified to explain how lncRNAs function *in vivo*. Accumulated lines of evidence suggest that very often lncRNAs function by recruiting and assembling other co-factors, which are usually proteins but possibly other RNAs [51,76,77] or DNAs [78]. Clearly, identifying these co-factors is of key importance for understanding lncRNA function.

The lncRNA *Xist* is capable of recruiting Polycomb Repressive Complex 2 (PRC2) to remodel chromatin modifications [79], resulting in transcriptional inactivation of one X chromosome. Similarly, *Air* and *Kcnq1ot1* lncRNAs achieve transcriptional silencing by recruiting chromatin-remodeling complexes during genomic imprinting [80,81]. Indeed, many lncRNAs have been identified to bind with PRC2 or other chromatin-modifying complexes for transcriptional repression [32,82]. In addition, lncRNAs can also activate gene transcription by binding specific protein factors. For instance, *Evf*-2 binds the Dlx-2 protein, which in turn increases the activity of the Dlx-5/6 enhancer [83]. Interestingly, one specific lncRNA might play complementary roles in gene expression regulation by selectively recruiting either PcG for repression [84] or Trithorax group proteins (TrxG) for activation [85]. 

In addition, lncRNAs can act as molecular scaffolds. For example, telomerase RNA component (TERC) acts as a flexible scaffold for bridging protein subunits together to promote telomerase activity [86]. *NEAT1* lncRNA is crucial for the integrity of paraspeckles [21,22,23,24], and a recent study revealed that *NEAT1* is capable of initiation of paraspeckle *de novo* formation [87]. 

Moreover, lncRNAs can also function as molecular sponges or decoys to affect gene regulation mediated by protein cofactors. For example, *Gas5* lncRNA binds the glucocorticoid receptor (GR) to compete against the association of the GR with other glucocorticoid response DNA elements, resulting in functional repression of GR [88]. PWS region *sno-lncRNA*s trap Fox family members to alter local Fox protein concentration and, subsequently, modulate Fox-regulated alternative splicing events [35]. Meanwhile, lncRNAs also act as competing endogenous decoys through their microRNA response elements (MREs) to titrate the availability of miRNAs for the other RNA molecules [30,51,76,77]. Finally, promoter associated lncRNAs can directly interact with enhancer DNA elements to form DNA:RNA triplexes to carry out their regulatory function [78].

Taken together, these studies suggest that the functional specificity of a given lncRNA is largely dependent on the association with its co-factors, mainly protein partners. Hence, it is important to find associated protein co-factors in order to fully understand the functional roles of lncRNAs. While the potential binding capacity can be predicted by computationally searching for consensus RNA sequences/motifs, direct lncRNA-protein interactomes can also be retrieved from cross-linking immuno-precipitation coupled with high-throughput sequencing (CLIP-seq) (Figure 1*b*), or using labeled lncRNAs as baits to pull down protein partners. 

How do lncRNAs bind to their protein co-factors? There are a variety of known mechanisms for this. *Xist* contains at least two distinct domains. One is the RepC domain, which is bound by YY1 and hnRNP U for the localization; the other one is the RepA domain, which recruits PRC2 for in-*cis* gene expression regulation [89,90]. Different from *Xist*, the PWS region *sno-lncRNA*s contain multiple consensus hexamer motifs for Fox family splicing regulators [91], which leads to the sequestration of Fox proteins and subsequently the alteration of patterns of Fox-regulated alternative splicing [35]. Interestingly, low evolutionarily conserved lncRNAs have been found associated with the same proteins. For example, human *NEAT1* and mouse *Men* *ε/β* share low primary sequence similarity, but both are associated with DBSH proteins [21,22,23,24]. This suggests that RNA structure features may sometimes play important roles in the determination of their protein partners. Thus, the recent application of genome-wide structural analysis that determines ncRNA secondary structure has begun to decipher the functional elements of the yeast transcriptome [92]. Similar studies in higher eukaryotes will help to reveal structural information and diverse biological insights of lncRNAs, possibly with their protein co-factors. 

## 4. Perspectives

In the era of post-genomics, elucidating the full spectrum of RNA molecules by a given cell is important for understanding gene expression and functional regulation. Largely from the previously imagined “dark matter” of the genome, a variety of lncRNAs have been systematically revealed from different tissues and species with clear characteristics distinguishing them from coding RNAs. The characteristics of lncRNAs are (1) low expression but with a pattern of tissue-specificity, (2) decreased conservation in primary sequence but with a likelihood of functional conservation, and (3) restrained coding capacity but with a probability of ancestral ORF relics. Transcriptome analyses by high-throughput technologies (including tiling arrays and RNA-seq) with high coverage, high sensitivity, and high efficiency represent an evolutionary leap in our methodology for lncRNA characterization. Recent studies have inspired new insights into the study of lncRNAs, and in turn, these insights have prompted further application of novel methodologies for lncRNA study.

Despite recent and rapid progress in our understanding of lncRNAs, a number of important features remain to be further addressed. For example, what are the landscapes of lncRNA expression in specific tissues/species and what are the connections of specific expression repertoires with specific tissue/species function? What are the distinct mechanisms for regulation of lncRNAs in specific tissues/species? What secondary structures are associated with lncRNA functions? Furthermore, existing computational algorithms are not sufficiently robust to deal with these sequence analyses. For example, they are less efficient for the accurate alignment of sequencing reads to lncRNAs in repetitive regions as well as for the precise transcript alignment of multiple lncRNA molecules from the same genomic segments.

Clearly, the integration of not only new computational pipelines, but also further experimental approaches, will be required to further our ability to discover new lncRNAs and how they function in gene regulation. 

## References

[1] International Human Genome Sequencing Consortium (2004). Finishing the euchromatic sequence of the human genome. Nature.

[2] Lander E.S., Linton L.M., Birren B., Nusbaum C., Zody M.C., Baldwin J., Devon K., Dewar K., Doyle M., FitzHugh W. (2001). Initial sequencing and analysis of the human genome. Nature.

[3] Yamada K., Lim J., Dale J.M., Chen H., Shinn P., Palm C.J., Southwick A.M., Wu H.C., Kim C., Nguyen M. (2003). Empirical analysis of transcriptional activity in the Arabidopsis genome. Science.

[4] Pennisi E. (2010). Shining a light on the genome's 'dark matter'. Science.

[5] Birney E., Stamatoyannopoulos J.A., Dutta A., Guigo R., Gingeras T.R., Margulies E.H., Weng Z., Snyder M., Dermitzakis E.T., Thurman R.E. (2007). Identification and analysis of functional elements in 1% of the human genome by the ENCODE pilot project. Nature.

[6] Clark M.B., Amaral P.P., Schlesinger F.J., Dinger M.E., Taft R.J., Rinn J.L., Ponting C.P., Stadler P.F., Morris K.V., Morillon A., Rozowsky J.S., Gerstein M.B., Wahlestedt C., Hayashizaki Y., Carninci P., Gingeras T.R., Mattick J.S.  (2011). The reality of pervasive transcription. PLoS Biol..

[7] Bartel D.P. (2009). MicroRNAs: target recognition and regulatory functions. Cell.

[8] Landgraf P., Rusu M., Sheridan R., Sewer A., Iovino N., Aravin A., Pfeffer S., Rice A., Kamphorst A.O., Landthaler M. (2007). A mammalian microRNA expression atlas based on small RNA library sequencing. Cell.

[9] Krzyzanowski P.M., Muro E.M., Andrade-Navarro M.A. (2012). Computational approaches to discovering noncoding RNA. RNA.

[10] Washietl S., Will S., Hendrix D.A., Goff L.A., Rinn J.L., Berger B., Kellis M. (2012). Computational analysis of noncoding RNAs. RNA.

[11] Chen L.L., Carmichael G.G.  (2010). Long noncoding RNAs in mammalian cells: what, where, and why?. RNA.

[12] Rinn J.L., Chang H.Y. (2012). Genome regulation by long noncoding RNAs. Annu. Rev. Biochem..

[13] Guttman M., Rinn J.L. (2012). Modular regulatory principles of large non-coding RNAs. Nature.

[14] Gibb E.A., Brown C.J., Lam W.L. (2011). The functional role of long non-coding RNA in human carcinomas. Mol. Cancer.

[15] Brown C.J., Ballabio A., Rupert J.L., Lafreniere R.G., Grompe M., Tonlorenzi R., Willard H.F. (1991). A gene from the region of the human X inactivation centre is expressed exclusively from the inactive X chromosome. Nature.

[16] Sleutels F., Zwart R., Barlow D.P. (2002). The non-coding Air RNA is required for silencing autosomal imprinted genes. Nature.

[17] Mancini-Dinardo D., Steele S.J., Levorse J.M., Ingram R.S., Tilghman S.M. (2006). Elongation of the Kcnq1ot1 transcript is required for genomic imprinting of neighboring genes. Genes Dev..

[18] Willingham A.T., Orth A.P., Batalov S., Peters E.C., Wen B.G., Aza-Blanc P., Hogenesch J.B., Schultz P.G. (2005). A strategy for probing the function of noncoding RNAs finds a repressor of NFAT. Science.

[19] Rinn J.L., Kertesz M., Wang J.K., Squazzo S.L., Xu X., Brugmann S.A., Goodnough L.H., Helms J.A., Farnham P.J., Segal E., Chang H.Y. (2007). Functional demarcation of active and silent chromatin domains in human HOX loci by noncoding RNAs. Cell.

[20] Hutchinson J.N., Ensminger A.W., Clemson C.M., Lynch C.R., Lawrence J.B., Chess A. (2007). A screen for nuclear transcripts identifies two linked noncoding RNAs associated with SC35 splicing domains. BMC Genomics.

[21] Chen L.L., Carmichael G.G. (2009). Altered nuclear retention of mRNAs containing inverted repeats in human embryonic stem cells: functional role of a nuclear noncoding RNA. Mol. Cell.

[22] Clemson C.M., Hutchinson J.N., Sara S.A., Ensminger A.W., Fox A.H., Chess A., Lawrence J.B. (2009). An architectural role for a nuclear noncoding RNA: NEAT1 RNA is essential for the structure of paraspeckles. Mol. Cell.

[23] Sasaki Y.T., Ideue T., Sano M., Mituyama T., Hirose T. (2009). MENepsilon/beta noncoding RNAs are essential for structural integrity of nuclear paraspeckles. Proc. Natl. Acad. Sci. USA.

[24] Sunwoo H., Dinger M.E., Wilusz J.E., Amaral P.P., Mattick J.S., Spector D.L. (2009). MEN epsilon/beta nuclear-retained non-coding RNAs are up-regulated upon muscle differentiation and are essential components of paraspeckles. Genome Res..

[25] Tripathi V., Ellis J.D., Shen Z., Song D.Y., Pan Q., Watt A.T., Freier S.M., Bennett C.F., Sharma A., Bubulya P.A. (2010). The nuclear-retained noncoding RNA MALAT1 regulates alternative splicing by modulating SR splicing factor phosphorylation. Mol. Cell.

[26] Zhang B., Arun G., Mao Y.S., Lazar Z., Hung G., Bhattacharjee G., Xiao X., Booth C.J., Wu J., Zhang C., Spector D.L. (2012). The lncRNA Malat1 is dispensable for mouse development but its transcription plays a cis-regulatory role in the adult. Cell Rep..

[27] Guttman M., Amit I., Garber M., French C., Lin M.F., Feldser D., Huarte M., Zuk O., Carey B.W., Cassady J.P. (2009). Chromatin signature reveals over a thousand highly conserved large non-coding RNAs in mammals. Nature.

[28] Cabili M.N., Trapnell C., Goff L., Koziol M., Tazon-Vega B., Regev A., Rinn J.L. (2011). Integrative annotation of human large intergenic noncoding RNAs reveals global properties and specific subclasses. Genes Dev..

[29] Chu C., Qu K., Zhong Franklin L., Artandi Steven E., Chang Howard Y.  (2011). Genomic Maps of Long Noncoding RNA Occupancy Reveal Principles of RNA-Chromatin Interactions. Mol. Cell.

[30] Cesana M., Cacchiarelli D., Legnini I., Santini T., Sthandier O., Chinappi M., Tramontano A., Bozzoni I. (2011). A long noncoding RNA controls muscle differentiation by functioning as a competing endogenous RNA. Cell.

[31] Wang K.C., Yang Y.W., Liu B., Sanyal A., Corces-Zimmerman R., Chen Y., Lajoie B.R., Protacio A., Flynn R.A., Gupta R.A. (2011). A long noncoding RNA maintains active chromatin to coordinate homeotic gene expression. Nature.

[32] Guttman M., Donaghey J., Carey B.W., Garber M., Grenier J.K., Munson G., Young G., Lucas A.B., Ach R., Bruhn L. (2011). LincRNAs act in the circuitry controlling pluripotency and differentiation. Nature.

[33] Pauli A., Rinn J.L., Schier A.F. (2011). Non-coding RNAs as regulators of embryogenesis. Nat. Rev. Genet..

[34] Yang L., Duff M.O., Graveley B.R., Carmichael G.G., Chen L.-L. (2011). Genomewide characterization of non-polyadenylated RNAs. Genome Biol..

[35] Yin Q.F., Yang L., Zhang Y., Xiang J.F., Wu Y.W., Carmichael G.G., Chen L.L. (2012). Long Noncoding RNAs with snoRNA Ends. Mol. Cell.

[36] Djebali S., Davis C.A., Merkel A., Dobin A., Lassmann T., Mortazavi A., Tanzer A., Lagarde J., Lin W., Schlesinger F. (2012). Landscape of transcription in human cells. Nature.

[37] Banfai B., Jia H., Khatun J., Wood E., Risk B., Gundling W.E., Kundaje A., Gunawardena H.P., Yu Y., Xie L. (2012). Long noncoding RNAs are rarely translated in two human cell lines. Genome Res..

[38] Derrien T., Johnson R., Bussotti G., Tanzer A., Djebali S., Tilgner H., Guernec G., Martin D., Merkel A., Knowles D.G. (2012). The GENCODE v7 catalog of human long noncoding RNAs: analysis of their gene structure, evolution, and expression. Genome Res..

[39] Struhl K. (2007). Transcriptional noise and the fidelity of initiation by RNA polymerase II. Nat. Struct. Mol. Biol..

[40] van Bakel H., Nislow C., Blencowe B.J., Hughes T.R. (2010). Most "dark matter" transcripts are associated with known genes. PLoS Biol..

[41] Johnson J.M., Edwards S., Shoemaker D., Schadt E.E. (2005). Dark matter in the genome: evidence of widespread transcription detected by microarray tiling experiments. Trends Genet..

[42] Guttman M., Garber M., Levin J.Z., Donaghey J., Robinson J., Adiconis X., Fan L., Koziol M.J., Gnirke A., Nusbaum C. (2010). Ab initio reconstruction of cell type-specific transcriptomes in mouse reveals the conserved multi-exonic structure of lincRNAs. Nat. Biotechnol..

[43] Mercer T.R., Dinger M.E., Sunkin S.M., Mehler M.F., Mattick J.S. (2008). Specific expression of long noncoding RNAs in the mouse brain. Proc. Natl. Acad. Sci. USA.

[44] Carninci P., Kasukawa T., Katayama S., Gough J., Frith M.C., Maeda N., Oyama R., Ravasi T., Lenhard B., Wells C. (2005). The transcriptional landscape of the mammalian genome. Science.

[45] Waterston R.H., Lindblad-Toh K., Birney E., Rogers J., Abril J.F., Agarwal P., Agarwala R., Ainscough R., Alexandersson M., An P. (2002). Initial sequencing and comparative analysis of the mouse genome. Nature.

[46] Ulitsky I., Shkumatava A., Jan C.H., Sive H., Bartel D.P. (2011). Conserved function of lincRNAs in vertebrate embryonic development despite rapid sequence evolution. Cell.

[47] Ponting C.P., Oliver P.L., Reik W. (2009). Mouse transcriptome: Evolution and functions of long noncoding RNAs. Cell.

[48] Hyashizaki Y. (2004). Mouse transcriptome: Neutral evolution of ‘non-coding’ complementary DNAs (reply). Nature.

[49] Wang J., Zhang J., Zheng H., Li J., Liu D., Li H., Samudrala R., Yu J., Wong G.K.  (2004). Neutral evolution of ‘non-coding’ complementary DNAs. Nature.

[50] Pang K.C., Frith M.C., Mattick J.S. (2006). Rapid evolution of noncoding RNAs: lack of conservation does not mean lack of function. Trends Genet..

[51] Marques A.C., Tan J., Lee S., Kong L., Heger A., Ponting C.P. (2012). Evidence for conserved post-transcriptional roles of unitary pseudogenes and for frequent bifunctionality of mRNAs. Genome Biol..

[52] Prasanth K.V., Spector D.L. (2007). Eukaryotic regulatory RNAs: an answer to the 'genome complexity' conundrum. Genes Dev..

[53] Esteller M. (2011). Non-coding RNAs in human disease. Nat. Rev. Genet..

[54] Dinger M.E., Pang K.C., Mercer T.R., Mattick J.S. (2008). Differentiating protein-coding and noncoding RNA: challenges and ambiguities. PLoS Comput. Biol..

[55] Okazaki Y., Furuno M., Kasukawa T., Adachi J., Bono H., Kondo S., Nikaido I., Osato N., Saito R., Suzuki H. (2002). Analysis of the mouse transcriptome based on functional annotation of 60,770 full-length cDNAs. Nature.

[56] Imanishi T., Itoh T., Suzuki Y., O'Donovan C., Fukuchi S., Koyanagi K.O., Barrero R.A., Tamura T., Yamaguchi-Kabata Y., Tanino M. (2004). Integrative annotation of 21,037 human genes validated by full-length cDNA clones. PLoS Biol..

[57] Duret L., Chureau C., Samain S., Weissenbach J., Avner P. (2006). The Xist RNA gene evolved in eutherians by pseudogenization of a protein-coding gene. Science.

[58] Kong L., Zhang Y., Ye Z.Q., Liu X.Q., Zhao S.Q., Wei L., Gao G. (2007). CPC: assess the protein-coding potential of transcripts using sequence features and support vector machine. Nucleic Acids Res..

[59] Altschul S.F., Madden T.L., Schaffer A.A., Zhang J., Zhang Z., Miller W., Lipman D.J. (1997). Gapped BLAST and PSI-BLAST: a new generation of protein database search programs. Nucleic Acids Res..

[60] Clamp M., Fry B., Kamal M., Xie X., Cuff J., Lin M.F., Kellis M., Lindblad-Toh K., Lander E.S. (2007). Distinguishing protein-coding and noncoding genes in the human genome. Proc. Natl. Acad. Sci. USA.

[61] Lin M.F., Jungreis I., Kellis M. (2011). PhyloCSF: a comparative genomics method to distinguish protein coding and non-coding regions. Bioinformatics.

[62] Cabianca D.S., Casa V., Bodega B., Xynos A., Ginelli E., Tanaka Y., Gabellini D. (2012). A long ncRNA links copy number variation to a polycomb/trithorax epigenetic switch in FSHD muscular dystrophy. Cell.

[63] Chen L.L., Carmichael G.G. (2010). Decoding the function of nuclear long non-coding RNAs. Curr. Opin. Cell Biol..

[64] Ingolia N.T., Lareau L.F., Weissman J.S. (2011). Ribosome profiling of mouse embryonic stem cells reveals the complexity and dynamics of mammalian proteomes. Cell.

[65] Kloc M., Wilk K., Vargas D., Shirato Y., Bilinski S., Etkin L.D. (2005). Potential structural role of non-coding and coding RNAs in the organization of the cytoskeleton at the vegetal cortex of Xenopus oocytes. Development.

[66] Candeias M.M., Malbert-Colas L., Powell D.J., Daskalogianni C., Maslon M.M., Naski N., Bourougaa K., Calvo F., Fahraeus R. (2008). P53 mRNA controls p53 activity by managing Mdm2 functions. Nat. Cell Biol..

[67] Mortazavi A., Williams B.A., McCue K., Schaeffer L., Wold B. (2008). Mapping and quantifying mammalian transcriptomes by RNA-Seq. Nat. Methods.

[68] Marzluff W.F., Wagner E.J., Duronio R.J. (2008). Metabolism and regulation of canonical histone mRNAs: life without a poly(A) tail. Nat. Rev. Genet..

[69] Wilusz J.E., Freier S.M., Spector D.L. (2008). 3' end processing of a long nuclear-retained noncoding RNA yields a tRNA-like cytoplasmic RNA. Cell.

[70] Cheng J., Kapranov P., Drenkow J., Dike S., Brubaker S., Patel S., Long J., Stern D., Tammana H., Helt G. (2005). Transcriptional maps of 10 human chromosomes at 5-nucleotide resolution. Science.

[71] Cui P., Lin Q., Ding F., Xin C., Gong W., Zhang L., Geng J., Zhang B., Yu X., Yang J., Hu S., Yu J. (2010). A comparison between ribo-minus RNA-sequencing and polyA-selected RNA-sequencing. Genomics.

[72] Gardner E.J., Nizami Z.F., Talbot C.C., Gall J.G. (2012). Stable intronic sequence RNA (sisRNA), a new class of noncoding RNA from the oocyte nucleus of Xenopus tropicalis. Genes Dev..

[73] Wilusz J.E., Jnbaptiste C.K., Lu L.Y., Kuhn C.D., Joshua-Tor L., Sharp P.A. (2012). A triple helix stabilizes the 3' ends of long noncoding RNAs that lack poly(A) tails. Genes Dev..

[74] Brown J.A., Valenstein M.L., Yario T.A., Tycowski K.T., Steitz J.A. (2012). Formation of triple-helical structures by the 3'-end sequences of MALAT1 and MENbeta noncoding RNAs. Proc. Natl. Acad. Sci. USA.

[75] Box J.A., Bunch J.T., Tang W., Baumann P. (2008). Spliceosomal cleavage generates the 3' end of telomerase RNA. Nature.

[76] Seitz H. (2009). Redefining microRNA targets. Curr. Biol..

[77] Salmena L., Poliseno L., Tay Y., Kats L., Pandolfi P.P. (2011). A ceRNA hypothesis: the Rosetta Stone of a hidden RNA language?. Cell.

[78] Schmitz K.M., Mayer C., Postepska A., Grummt I. (2010). Interaction of noncoding RNA with the rDNA promoter mediates recruitment of DNMT3b and silencing of rRNA genes. Genes Dev..

[79] Zhao J., Sun B.K., Erwin J.A., Song J.J., Lee J.T. (2008). Polycomb proteins targeted by a short repeat RNA to the mouse X chromosome. Science.

[80] Nagano T., Mitchell J.A., Sanz L.A., Pauler F.M., Ferguson-Smith A.C., Feil R., Fraser P. (2008). The Air noncoding RNA epigenetically silences transcription by targeting G9a to chromatin. Science.

[81] Pandey R.R., Mondal T., Mohammad F., Enroth S., Redrup L., Komorowski J., Nagano T., Mancini-Dinardo D., Kanduri C. (2008). Kcnq1ot1 antisense noncoding RNA mediates lineage-specific transcriptional silencing through chromatin-level regulation. Mol. Cell.

[82] Khalil A.M., Guttman M., Huarte M., Garber M., Raj A., Rivea Morales D., Thomas K., Presser A., Bernstein B.E., van Oudenaarden A. (2009). Many human large intergenic noncoding RNAs associate with chromatin-modifying complexes and affect gene expression. Proc. Natl. Acad. Sci. USA.

[83] Feng J., Bi C., Clark B.S., Mady R., Shah P., Kohtz J.D. (2006). The Evf-2 noncoding RNA is transcribed from the Dlx-5/6 ultraconserved region and functions as a Dlx-2 transcriptional coactivator. Genes Dev..

[84] Schmitt S., Prestel M., Paro R. (2005). Intergenic transcription through a polycomb group response element counteracts silencing. Genes Dev..

[85] Sanchez-Elsner T., Gou D., Kremmer E., Sauer F. (2006). Noncoding RNAs of trithorax response elements recruit Drosophila Ash1 to Ultrabithorax. Science.

[86] Zappulla D.C., Cech T.R. (2004). Yeast telomerase RNA: a flexible scaffold for protein subunits. Proc. Natl. Acad. Sci. USA.

[87] Mao Y.S., Sunwoo H., Zhang B., Spector D.L. (2011). Direct visualization of the co-transcriptional assembly of a nuclear body by noncoding RNAs. Nat. Cell Biol..

[88] Kino T., Hurt D.E., Ichijo T., Nader N., Chrousos G.P. (2010). Noncoding RNA gas5 is a growth arrest- and starvation-associated repressor of the glucocorticoid receptor. Sci. Signal.

[89] Wutz A., Rasmussen T.P., Jaenisch R. (2002). Chromosomal silencing and localization are mediated by different domains of Xist RNA. Nat. Genet..

[90] Hasegawa Y., Brockdorff N., Kawano S., Tsutui K., Nakagawa S. (2010). The matrix protein hnRNP U is required for chromosomal localization of Xist RNA. Dev. Cell.

[91] Yeo G.W., Coufal N.G., Liang T.Y., Peng G.E., Fu X.-D., Gage F.H. (2009). An RNA code for the FOX2 splicing regulator revealed by mapping RNA-protein interactions in stem cells. Nat. Struct. Mol. Biol..

[92] Wan Y., Qu K., Ouyang Z., Kertesz M., Li J., Tibshirani R., Makino D.L., Nutter R.C., Segal E., Chang H.Y. (2012). Genome-wide Measurement of RNA Folding Energies. Mol. Cell.

